# Supplemental methionine selenium effects on egg yolk physicochemical, functional, and protein structure during storage

**DOI:** 10.3389/fnut.2023.1207754

**Published:** 2023-06-05

**Authors:** Dan Chen, Yaotong Liu, Shiwen Xu

**Affiliations:** ^1^College of Veterinary Medicine, Northeast Agricultural University, Harbin, China; ^2^College of Food Science, Northeast Agricultural University, Harbin, China

**Keywords:** methionine selenium, egg yolk, storage, gelation, surface tension

## Abstract

To clarify the effect of the addition of methionine selenium on the physicochemical, functional, and protein structural properties of egg yolk during storage. We analyzed the changes in the main indicators of egg yolks stored at 4°C and 25°C for 28 days. The results showed that the increase in water content and pH, and the decrease in absolute zeta potential and apparent viscosity of the selenium-rich egg yolks (Se-group) during storage were smaller than those of the control group egg yolks (C-group). In addition, the antioxidant capacity and emulsifying ability of the Se-group during storage were better than those of the C-group. Simultaneously, the hardness and chewiness of the Se-group gel during storage were lower than those of the C-group. The protein structure results showed that selenium rich treatment did not affect the secondary structure of egg yolk protein during storage but could improve the fluorescence intensity of the egg yolk protein. Therefore, the addition of methionine selenium can reduce the degree of deterioration in the physicochemical properties of egg yolk during storage and extend its shelf life.

## 1. Introduction

With the development of science and technology and changes in consumer preferences, the structure of egg products in China is constantly diversified. Eggs are one of the most important sources of food protein in people’s daily lives and can also serve as an ideal carrier for providing organic selenium functional foods (1). Previous studies have shown that mixing different forms of selenium products into chicken feed during daily feeding can significantly increase the selenium content in eggs (2). Among them, supplementing selenium in the form of methionine selenium will cause more selenium to precipitate into egg white (3). Furthermore, increased selenium content in eggs is manifested by a stronger antioxidant protective effect of egg yolk, longer egg stability, and increased nutritional value (4). Therefore, selenium-rich eggs have great potential as nutritional supplements to effectively prevent malnutrition among vulnerable groups.

Eggs are living substances, and their quality gradually decreases after leaving the mother’s body, especially under the influence of temperature. Egg yolk, as one of the main components in eggs, is rich in fat, minerals, vitamins, and proteins (5). The egg yolk index of normal eggs ranges from 0.38 to 0.44, while the egg yolk index of fresh eggs is greater than 0.4 and that of qualified eggs is 0.3–0.35. When it is less than 0.25, the egg yolk membrane ruptures. The egg yolk index gradually drops during storage as a result of the water in the egg white permeating the yolk and the salts in the yolk permeating the white (6). The strength of the egg yolk membrane is the main factor in producing high-quality egg white. During long-term storage, the egg yolk membrane is affected by factors such as egg yolk volume and has weak springiness, which can easily cause rupture (7). Xiong et al. studied the relationship between egg freshness and storage time and found that egg weight and egg yolk coefficient significantly decreased with storage time (8). Du et al. studied the variation in egg freshness with storage conditions and found that with prolonged storage time, the egg weight loss rate and air height increased, while the Huff unit and egg yolk index decreased (9). Yu and Wang found that the quality and yolk coefficient of eggs decrease with prolonged storage time, while the air height increases with prolonged storage time. The kinetic reaction rate constant indicates that the higher the storage temperature is, the more significant the decrease in egg quality (10). At present, research on the changes in eggs during storage mainly focuses on the evaluation of egg freshness or the phenomenon of egg white thinning, while there are relatively few reports on the changes in egg yolk during storage (1). It takes approximately 8–12 days for an egg to be born and sold, during which time it has already been affected by external environmental factors, resulting in a decrease in its internal quality and shortened storage time. Research has found that the storage time of eggs produced by feeding organic selenium is relatively long (11). Enriched selenium can alleviate the decrease in Huff units during egg storage, and the effect of organic selenium is better than that of inorganic selenium. Compared with inorganic selenium, organic selenium is more likely to participate in the metabolic process of substances in the body, extending the shelf life of eggs (12, 13). However, there is relatively little research on the changes in the yolk of selenium-rich eggs during storage.

We used selenium methionine (SeMet) as a selenium source material and fed it for 42 days to obtain selenium-rich eggs. Previous studies found that adding SeMet increased the egg production rate, average egg weight, trace element and total amino acid content of laying hens, reduced the shell breaking rate, and improved the composition of fatty acids in eggs. To evaluate the impact of methionine selenium further comprehensively on egg quality, we studied the changes in the physicochemical properties, functional characteristics, and protein structural characteristics of egg yolks stored at 4°C and 25°C for 28 days, which is of great significance for improving the development and utilization of selenium-rich eggs.

## 2. Materials and methods

### 2.1. Materials and reagents

Eggs were obtained from the laboratory, and ANS was purchased from Solaibao Co., Ltd. (Beijing, China), superoxide dismutase activity test kit (SOD), malondialdehyde content test kit (MDA), total antioxidant capacity test kit (T-AOC), catalase test kit (CAT), and glutathione peroxidase test kit (GPx) were purchased from Nanjing Jiancheng Bioengineering Institute (Nanjing, China).

### 2.2. Sample collection

In this experiment, 48 week-old Hailan brown laying hens were selected, and all laying hens were raised in the same coop and adapted to the environment for 7 days. Sixty hens were randomly divided into two groups (30 per group): a control group (basal diet) and a SeMet group (basal diet supplemented with SeMet at a concentration of 0.75 mg/kg), fed for 42 days. The basic diet consists of 40% concentrated feed and 60% corn. The composition of concentrated feed include 30% crude protein, 9% crude fiber, 38% crude ash, 6 ~ 12% calcium, 0.6% phosphorus, 0.3 ~ 2% sodium chloride, and 0.6% methionine. It is given 16 h of light per day, and the temperature inside the house is maintained between 20–23°C. Eggs were collected after 6 weeks of feeding. The collected eggs were randomly divided into two groups and stored at 4°C and 25°C for 28 days, with sampling and measurement conducted every 7 days. Water and food can be freely consumed. Conventional vaccination procedures were used. All laying hens maintained good health during the feeding period. This study was conducted in accordance with the “Guidelines for the Care and Use of Experimental Animals” issued by Northeast Agricultural University. This experimental plan was approved by the Animal Experiment Ethics Committee of Northeast Agricultural University.

### 2.3. Determination of water content

The water content was determined according to the method in GB 5009.3-2019 (China). Place the aluminum foil weighing plate in an oven in advance, heat it to constant weight at 105°C, and record the weight of the aluminum foil weighing plate as *m*_1_. Subsequently, weigh a sample greater than 5.00 g (record the weight of the sample as *m*_2_) onto an aluminum foil weighing plate and heat it to constant weight at 105°C. Finally, the sample and aluminum foil weighing plate were placed in a dryer to room temperature, and the weight was recorded in *m*_3_. The water content in the egg yolk was recorded according to the following formula:


$$\mathrm{Water content}\;\left(\%\right)=\frac{m_{2}-\left(m_{3}-m_{1}\right)}{m_{2}}$$

### 2.4. Determination of pH

The egg yolk was placed in a beaker after breaking, and the pH value of the sample was measured directly with an electronic pH meter (PHS-3C, Shanghai Precision Instruments Co., Ltd., Shanghai, China).

### 2.5. Determination of egg yolk index

Vernier calipers (500-151-30, Mitutoyo, Kawasaki, Japan) were used to measure the diameter and height of egg yolks. Each egg was measured three times at points separated by a rotation of 120°, and the average of the three measurements was calculated.


$$\mathrm{Egg}\;\mathrm{yolk index}=\frac{\mathrm{Height of}\;\mathrm{egg}\;\mathrm{yolk}}{\mathrm{Diameter of}\;\mathrm{egg}\;\mathrm{yolk}}$$

### 2.6. Determination of zeta potential

The zeta potential was determined using the method described by Liu et al. (14) with slight modifications. The sample was diluted 500 times with deionized water and stirred with magnetic force until the sample was evenly mixed for measurement. The measurement conditions are as follows: the colorimetric cell specification is a 1 cm polystyrene cell, using a pair of 0.45 cm^2^ platinum electrodes with a spacing of 0.4 cm, the measured temperature is 25°C, the temperature equilibrium time is 2 min, and each group includes 3 measurements.

### 2.7. Determination of rheological properties

The rheological properties of egg yolks were determined using a HAAKE MARS40 rheometer (Thermo Fisher Scientific, Massachusetts, United States) in the dynamic rheometer shear mode. Place the sample between the parallel plates of the rheometer, and carefully remove the excess using a razor. A flat plate with a diameter of 35 mm and a gap distance of 0.3 mm was used. The shear rate scanning parameters were as follows: temperature 25°C and shear rate 0.1–100 s^−1^.

With a fixed strain value of 1% and a plate clearance of 0.3 mm, the dynamic oscillatory frequency sweep was determined for dynamic viscoelastic measurements over a frequency range of 0.1–16 Hz at 25 ± 1°C. After gathering the *G*′ and *G*″ values from this test, the loss tangent (tan*δ*) was calculated using the formula shown below:


$$\tan\;\delta =\frac{G^{\prime \prime }}{G^{\prime }}$$

### 2.8. Determination of gel properties

#### 2.8.1. Preparation of egg yolk gel

The egg yolk transfer value square ice cube mold (16 mm × 16 mm) was placed in a water bath pot and heated in a 90°C water bath for 30 min to prepare the protein gel. Take out gel, cool it in ice water to room temperature, and place in a 4°C refrigerator for 20–24 h.

#### 2.8.2. Texture property measurements

The gel strength was measured using a TPA texture analyzer (TA.new plus, ISENSO, New York, NY, United States). The sample was measured at 25°C. The operating conditions of the instrument were as follows: using a P36R probe, the speed before the test was 2.0 mm/s, the test speed was 1.0 mm/s, the speed after the test was 2.0 mm/s, the triggering force was 10 g, the degree of deformation was 40%, and the interval time was 5.0 s.

#### 2.8.3. Magnetic resonance imaging measurements

With reference to the techniques of Liu et al. (1), a low-field nuclear magnetic resonance analyzer (MesoMR23-060H-I, Suzhou Niumag Co., Ltd., China) was used analyze the proton density of egg yolk gel.

### 2.9. Determination of surface tension

Egg yolk was diluted in a 1:500 solution with deionized water. Using the drop-drop technique in the DSA25S instrument (KRUSS, Germany), the interfacial tension between egg yolk solution and soybean oil over time was measured under various storage conditions. In a 25 mL colorimetric dish filled with soybean oil, egg yolk dilution droplets were generated using a syringe with an outer diameter of 1.059 mm, and the interfacial tension was measured. Fifteen microliter droplets were prepared at an output rate of 30 μL/s. The computer automatically collects and detects images quickly and fits the Young Laplace’s equation to determine the interfacial tension.

### 2.10. Determination of antioxidant capacity

The superoxide dismutase activity (SOD), malondialdehyde content (MDA), total antioxidant capacity (T-AOC), catalase (CAT), and glutathione peroxidase (GPx) were determined with a kit from Nanjing Jiancheng Bioengineering Research Institute. The steps were completed according to the instructions.

### 2.11. Fourier transform infrared spectroscopic measurements (FTIR)

An FTIR spectrometer (Nicolet iS 10, Thermo Fisher Scientific Co., Ltd., Waltham, United States) was used to record the change in the sample spectrum. The instrument parameters were as follows: scanning resolution, 4 cm^−1^; scanning range, 4,000–400 cm^−1^.

### 2.12. Fluorescence spectroscopy measurements

A fluorescence spectrophotometer (F-7100, Hitachi Limited, Tokyo, Japan) was used to measure fluorescence using the method of Jiang et al. (15) with a few minor modifications. Egg yolk protein concentrations were adjusted to 1 mg/mL with 0.01 mol/L PBS (pH 7.0). Excitation wavelength: 280 nm, emission wavelength: 300–420 nm, slit width: 5 nm, and scanning speed: 60 nm/min are the measurement parameters.

### 2.13. SDS–PAGE

The composition of egg yolk protein was analyzed using Laemmli’s (16) method, with a 4% stacking gel and a 12% separating gel and an additional sample size of 10 μL. Make a solution with a protein content of 2 mg/mL from the sample.

### 2.14. Statistical analysis

The analysis values are expressed as the means ± SDs. One-way ANOVA was used for the statistical analysis in the SPSS 19.0 program. Using Duncan’s multiple-range tests for means with a 95% confidence limit (*p* < 0.05), the mean differences were calculated. The differences between C-group and Se-group using Student’s t-test.

## 3. Results and discussion

### 3.1. FITR analysis

**Figure 1:**
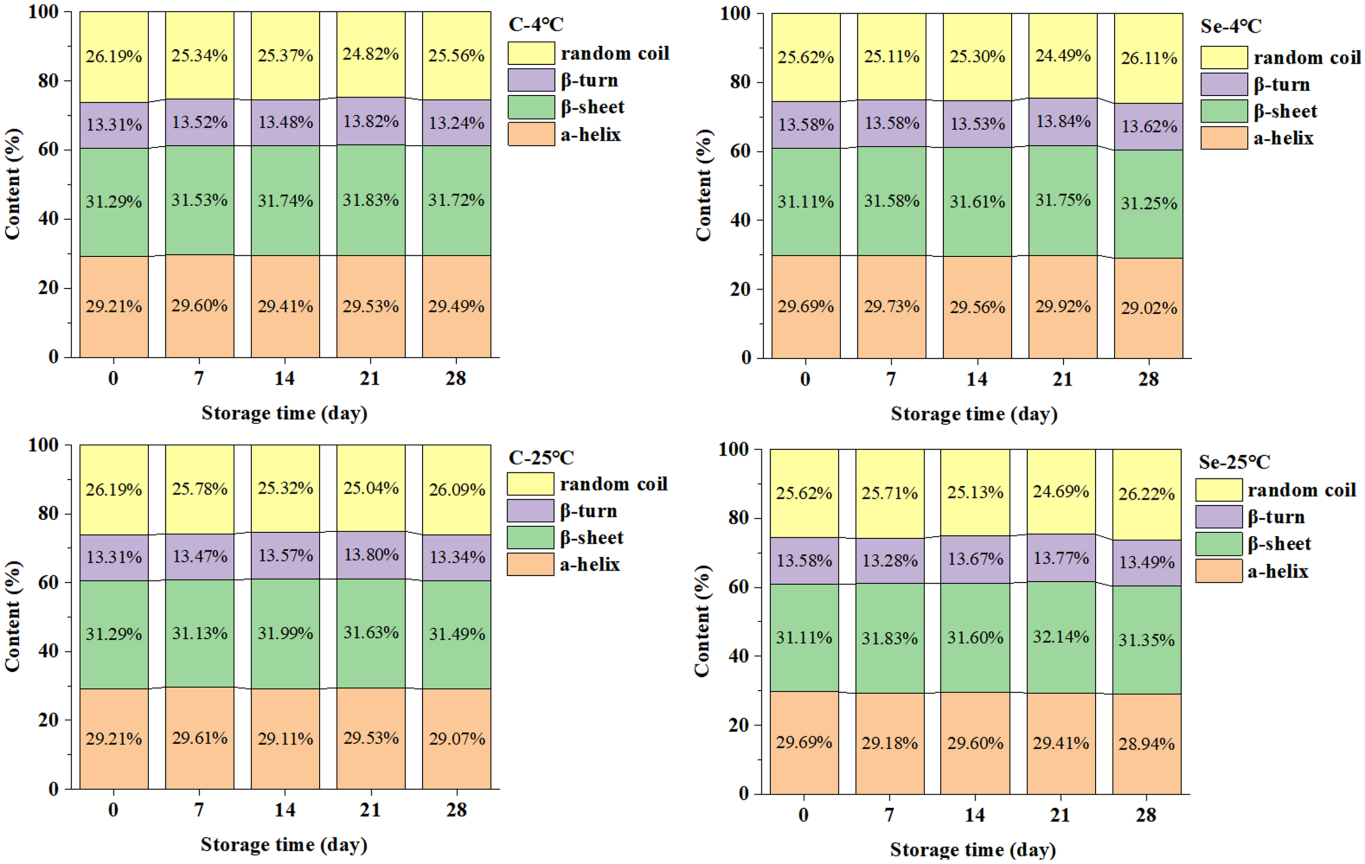
Changes in secondary structure of egg yolk protein during storage at 4°C and 25°C.

Proteins are biological macromolecules with specific structures formed by connecting amino acids through skin bonds. The main active groups are peptide bonds, aromatic amino acid residues, and disulfide bonds in the skin chain skeleton (17). The changes in the secondary structure content of protein in egg yolk during storage are shown in Figure 1. The secondary structure of egg yolk protein is mainly composed of α-helices and β-sheets (accounting for over 60% of the total), with relative contents of 29 and 31%, respectively. In addition, the content of β-turns is 29%, and the content of irregular curls is 26%.

During storage at 4°C, α-helix in the Se-group decreased from 29.69 to 29.02%, β-sheet increased from 31.11 to 31.25%, β-turn increased from 13.58 to 13.62%, and irregular curls increased from 25.62 to 26.11%; α-helix in the C-group increased from 29.21 to 29.49%, β-sheet increased from 31.29 to 31.72%, β-turn increased from 13.31 to 13.24%, and irregular curls decreased from 26.19 to 25.56%. During storage at 25°C, α-helix in the Se-group decreased from 29.69 to 28.94%, β-sheet increased from 31.11 to 31.35%, β-turn decreased from 13.58 to 13.49%, and irregular curls increased from 25.62 to 26.22%; α-helix in the C-group decreased from 29.21 to 29.07%, β-sheet increased from 31.29 to 31.49%, β-turn increased from 13.31 to 13.34%, and irregular curls decreased from 26.19 to 26.09%. Compared to the C-group, the Se-group had a higher relative content of α-helix in egg yolk protein, indicating that selenium-rich treatment can slow the transition of egg yolk protein to disorder. In addition, the increase in β-sheet content will enhance the interaction of protein, protein-lipid, protein-water and promote the formation of a protein gel network (18). The results showed that the content of β-sheets in egg yolk protein in the Se-group was lower than that in the C-group. Therefore, we speculate that egg yolk aggregation occurs during storage, and selenium-rich treatment can slow the collision between proteins.

### 3.2. Fluorescence spectroscopy analysis

**Figure 2:**
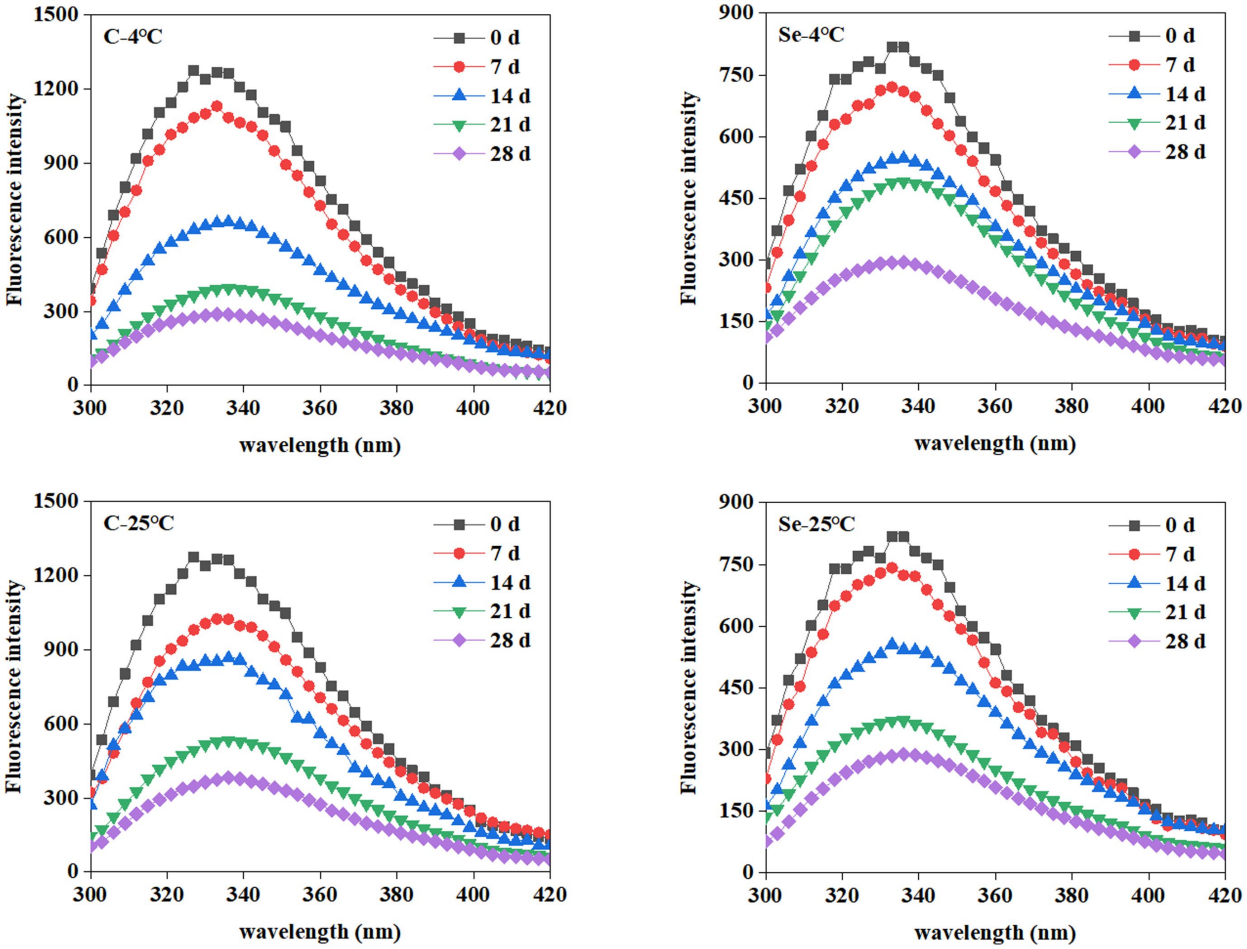
Changes of fluorescence spectrum of egg yolk during storage at 4°C and 25°C.

Due to high sensitivity, selectivity, and broad dynamic range, fluorescence spectroscopy has been extensively used in protein analysis. The aromatic amino acids (Trp, Tyr, and Phe) in proteins can be used as fluorescent probes to research the conformation, structure, and function of proteins (19). Figure 2 shows the changes in the fluorescence spectra of egg yolk protein during storage at 4°C and 25°C. The maximum fluorescence intensity of the endogenous fluorescence spectrum of the C-group and Se-group decreases with the extension of storage time during different temperature storage periods. In addition, the effect of storage at 4°C and 25°C on the endogenous fluorescence spectrum of egg yolks was basically the same, while the maximum fluorescence intensity of the endogenous fluorescence spectrum of the Se-group was always lower than that of the C-group.

The decrease in the fluorescence intensity of egg yolk during storage indicates protein aggregation during the storage process, thereby reducing the output of Trp residue fluorescence emission energy and leading to a decrease in the fluorescence intensity of the endogenous fluorescence spectrum. Karoui et al. found that the fluorescence intensity of the endogenous fluorescence spectrum of egg yolk decreased during 29 days of storage at room temperature, indicating that Trp residues were in an increasingly hydrophobic microenvironment (20). Egg yolk is a natural emulsification system, while Rampon et al. explored the endogenous fluorescence spectrum of oil in water lotion prepared from bovine serum albumin during storage, and the results showed that the reduction in the fluorescence intensity of its endogenous fluorescence spectrum during storage may also be caused by the degradation of amino acid residues during storage (21). It is worth noting that the decrease in the maximum fluorescence intensity of the endogenous fluorescence spectrum of the Se group during the entire storage period was lower than that of the C-group, indicating that Se-group egg yolks are more tolerant to storage.

### 3.3. SDS–PAGE analysis

**Figure 3:**
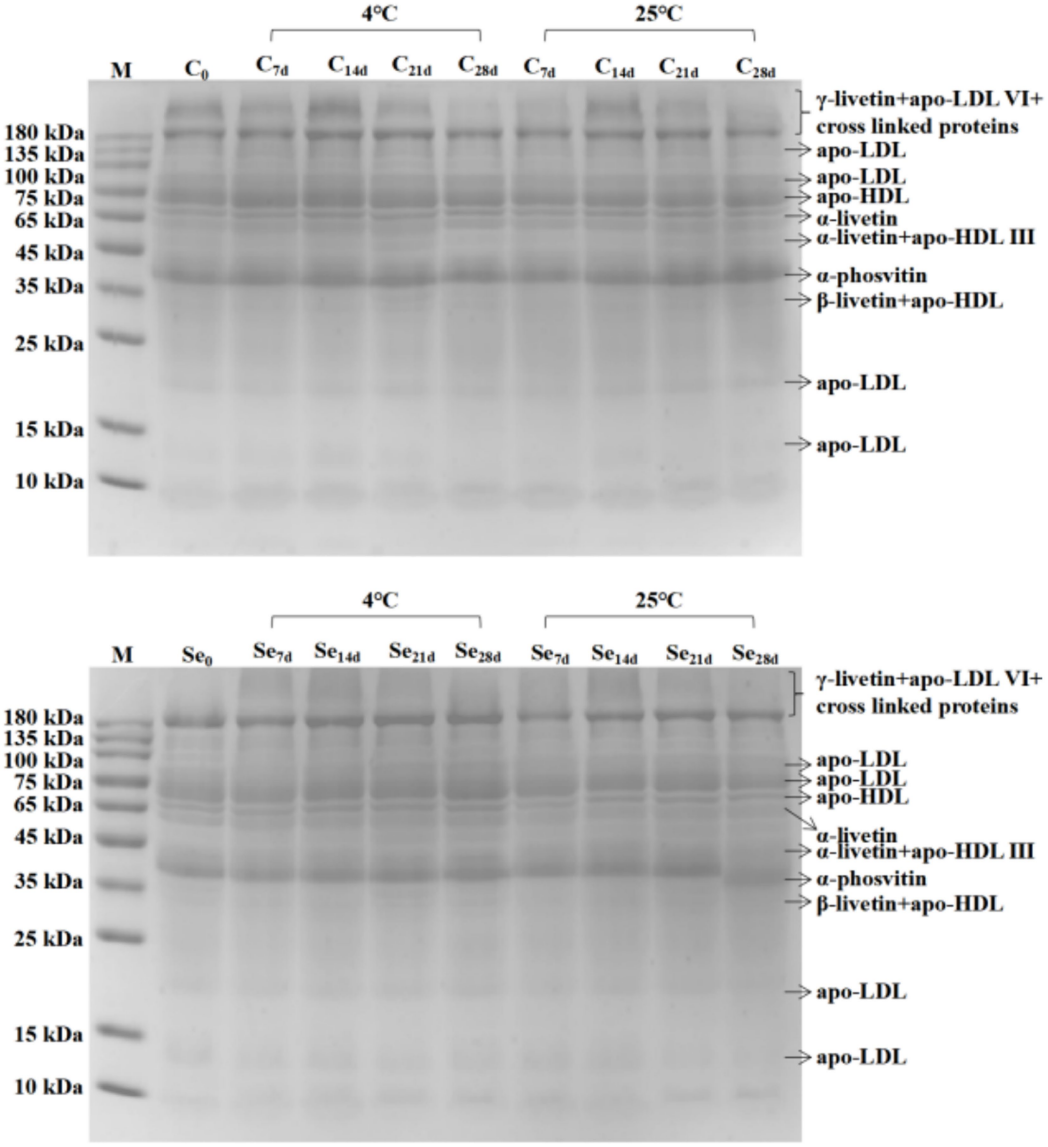
SDS–PAGE analysis of egg yolk total protein SDS–PAGE during storage at 4°C and 25°C.

Egg yolk is a high-quality source of protein, and changes in protein during storage can also have adverse effects on its quality. SDS–PAGE of egg yolk protein during storage at 4°C and 25°C is shown in Figure 3. Compared with the C-group, the protein bands of the Se-group at 200–220 kDa were not significant, the protein abundance at 180 kDa was higher, and the protein abundance at 10 kDa was lower. The changes in other protein abundances were not significant. According to the results of different storage temperatures, the protein abundance at 180 kDa and 73 kDa in the C-group and Se-group at 4°C was higher than that in the egg yolk protein at 25°C. The C-group showed significant protein bands between 200 ~ 220 kDa, while the Se-group did not show any protein bands within this range. However, there was no difference in the composition of egg yolk protein in the same group under storage conditions of 4°C and 25°C. Meanwhile, there was also no difference in protein composition between the C-group and Se-group.

### 3.4. Changes in water content, pH value, and egg yolk index

**Figure 4:**
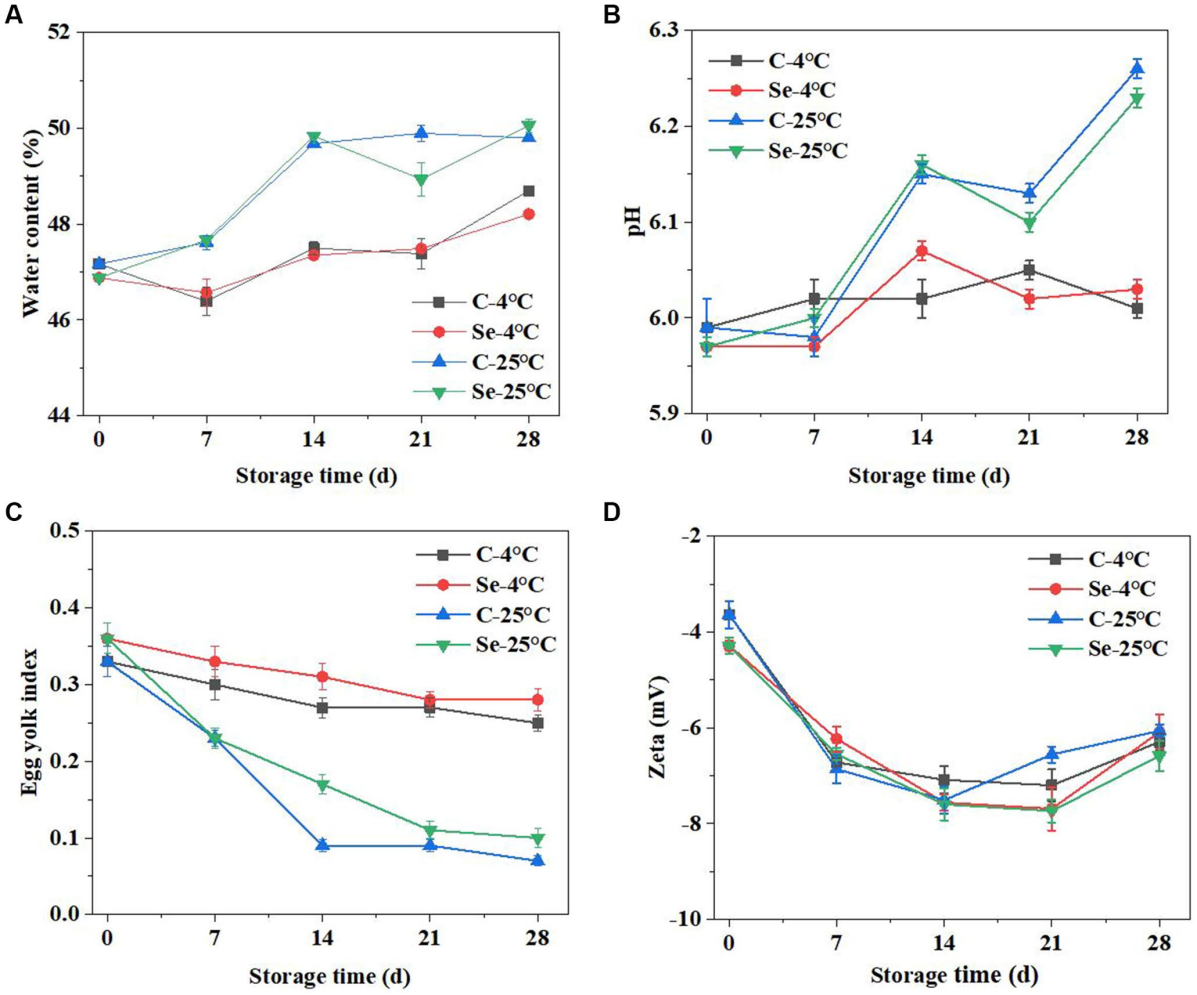
Changes in water content **(A)**, pH value **(B)**, egg yolk index **(C)**, and Zeta potential **(D)** of egg yolk during storage at 4°C and 25°C.

The changes in water content in the egg yolks of the Se group and C group at storage temperatures of 4°C and 25°C are shown in Figure 4A. The significant change in egg yolk during storage is the phenomenon of egg yolk filling with water. As the storage time increases, the water content of all egg yolks also increases, which may be caused by the transfer of water from egg white to egg yolk (22). The increase in egg yolk water content at 4°C was significantly slower than that at 25°C (*p* < 0.05), indicating that low temperature can inhibit the transfer of water from egg white to egg yolk. Comparing the water content of egg yolks between the C-group and Se-group, it can be concluded that selenium enrichment may reduce the transfer of egg white water to the egg yolk. Comparing the results of egg yolk at 4°C and 25°C, the increase in egg yolk water content at 4°C was significantly slower than that at 25°C (*p* < 0.05). Liu found that 4°C can effectively inhibit the respiratory intensity of eggs, which is beneficial for maintaining egg quality (23). Under the action of osmotic pressure, water is constantly transferred to the egg yolk through the egg yolk membrane. The increased water will stretch the egg yolk membrane, resulting in the egg yolk flattening during storage and the egg yolk index declining. An increase in storage temperature will increase the water content in the egg yolk, weaken the strength of the egg yolk membrane, and make the egg yolk fragile (24). Furthermore, the disulfide bonds of glycoproteins outside the egg yolk membrane and ovomucin progressively degrade during storage, resulting in a decrease in egg yolk membrane strength (25). Research has shown that selenium enrichment can enhance the strength of egg yolk membranes, which helps to reduce damage to egg yolks during transportation or beating, as well as water migration, and is beneficial for improving the quality of shell eggs (26, 27).

The change in pH value is an important indicator of the freshness of eggs, and the (Figure 4B) shows the changes in the pH value of egg yolks in the C-group and Se-group. The pH value of egg yolk significantly increased with increasing storage time. The pH of egg yolk during storage is negatively correlated with the strength of the egg yolk membrane, which may be due to protein degradation. During the degradation process of protein, concentrated ammonia is produced, leading to an increase in pH (28). Comparing the results of 4°C and 25°C, the pH value at 4°C was significantly lower than that at 25°C, and the pH of the egg yolk in the Se group was lower than that in the C-group. The degradation of proteins is related to lipid oxidation that occurs during egg yolk storage. However, due to the presence of selenium, the oxidation stability of Se-group egg yolks is higher than that of C-group egg yolks. Therefore, during both storage processes, the oxidation degree of the Se group is lower than that of the C group, indicating that Se-group egg yolks are more resistant to storage.

The egg yolk index refers to the ratio of egg yolk height to egg yolk diameter and is often used to indicate the freshness of an egg. A smaller egg yolk index indicates a lower freshness of the egg. As shown in Figure 4C, under storage conditions of 4°C and 25°C, the egg yolk index showed varying degrees of decline. At a storage time of 7 days, the egg yolk index under storage at 25°C was significantly lower than that at 4°C. Observing the Se-group and C-groups, under storage conditions of 4°C and 25°C, the egg yolk index of the Se-group is higher than that of the C-group at 14 days, indicating that selenium rich can maintain the freshness of eggs under certain conditions.

### 3.5. Changes in zeta potential

The zeta potential can be used to assess colloidal dispersion system stability and to determine the degree of electrostatic repulsion and interaction between protein molecules (29). Figure 4D indicates that the zeta potential absolute values of Se-group and C-group egg yolks showed a trend of first increasing and then decreasing during storage, which indicates that the surface charge of egg yolk increases during storage, increasing the potential of the protein and reducing its adsorption rate. Selenium-rich eggs can reduce cholesterol in egg yolks, and compared to regular eggs, the difference in zeta potential values between the two is relatively small (30). During storage, some enzymes in the egg yolk decompose organic macromolecules, leading to a decrease in the concentration of the egg yolk content, thereby increasing the tension borne by the egg yolk membrane, leading to the rupture of the egg yolk membrane and egg yolk dispersion (31, 32). The composition of egg yolk protein changes during this period, resulting in changes in the zeta potential value. In the later stage of storage (14–28 days), the absolute value of the zeta potential of the egg yolk decreases, which may be due to the interaction between protein molecules reducing the adsorption potential energy at the oil–water interface, leading to a decrease in the stability of the system. Under 25°C storage conditions, the absolute decrease in the zeta potential of the Se-group was lower than that of the C-group, indicating that the stability of Se-group egg yolks was better than that of the C-group during storage, further proving the high storage stability of selenium-rich egg yolks.

### 3.6. Changes in rheological properties

**Figure 5:**
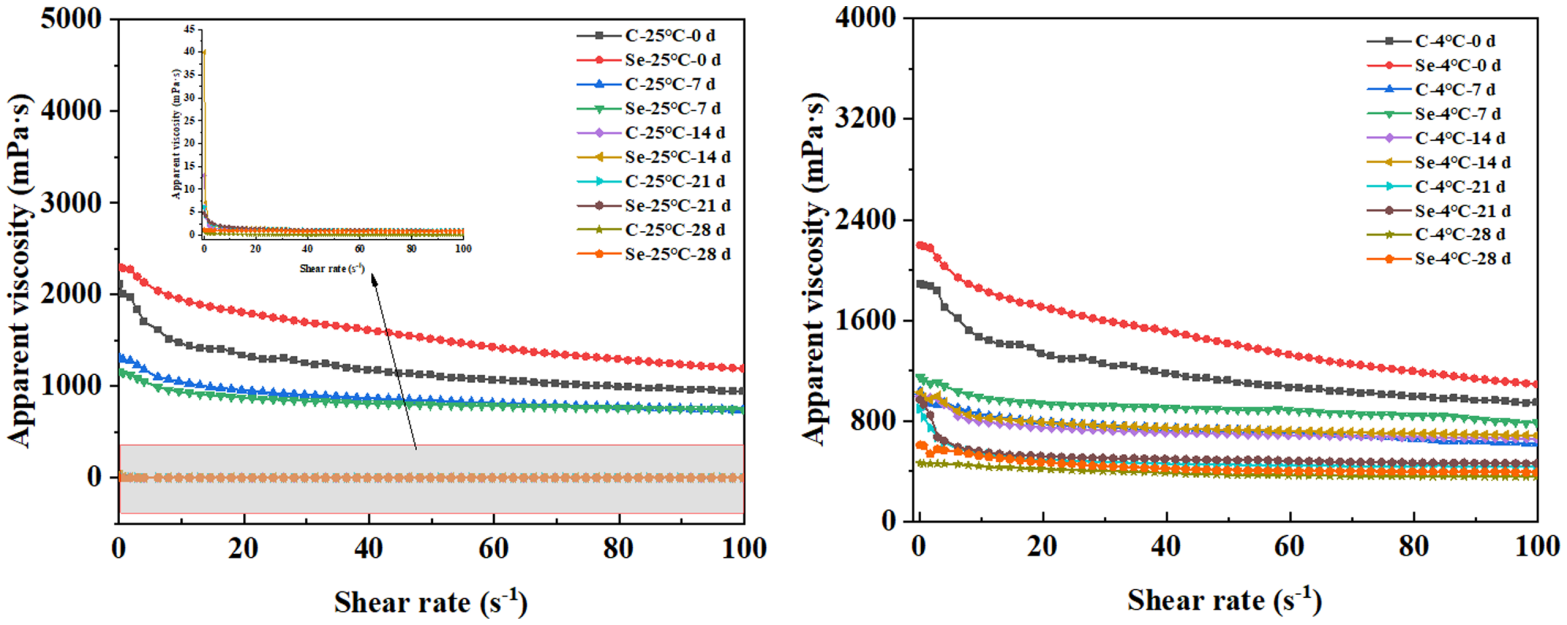
Changes in apparent viscosity of egg yolk during storage at 4°C and 25°C.

Another phenomenon caused by water filling of egg yolk is egg yolk thinning. This project aims to provide a reference to produce egg gel products through the rheological properties of egg yolk. The changes in the apparent viscosity of the Se-group and C-group under different storage temperatures and times are shown in Figure 5. With increasing shear force, the apparent viscosity of all egg yolk samples decreased to varying degrees, indicating a shear thinning phenomenon. This result indicates that egg yolk belongs to a pseudoplastic fluid. Meanwhile, as the storage temperature increases, the apparent viscosity of the egg yolk decreases, exhibiting a significant thinning phenomenon. Kumbár et al. (33) and Severa et al. (27) also observed similar results. The decrease in stability and improvement in fluidity of egg yolk samples may be due to an increase in storage time leading to a decrease in the egg yolk content and changes in the tension of the egg yolk membrane causing egg yolk dispersion. However, under the same storage temperature and time, the degree of decrease in apparent viscosity of the Se-group samples was significantly smaller than that of the C-group, indicating that SeMet can to some extent suppress the problem of decreased egg yolk viscosity during storage and maintain the original quality of the egg yolk. The possible reason for this change is that SeMet reduces the cholesterol content in egg yolk, further enhancing the interaction between molecules in egg yolk.

**Figure 6:**
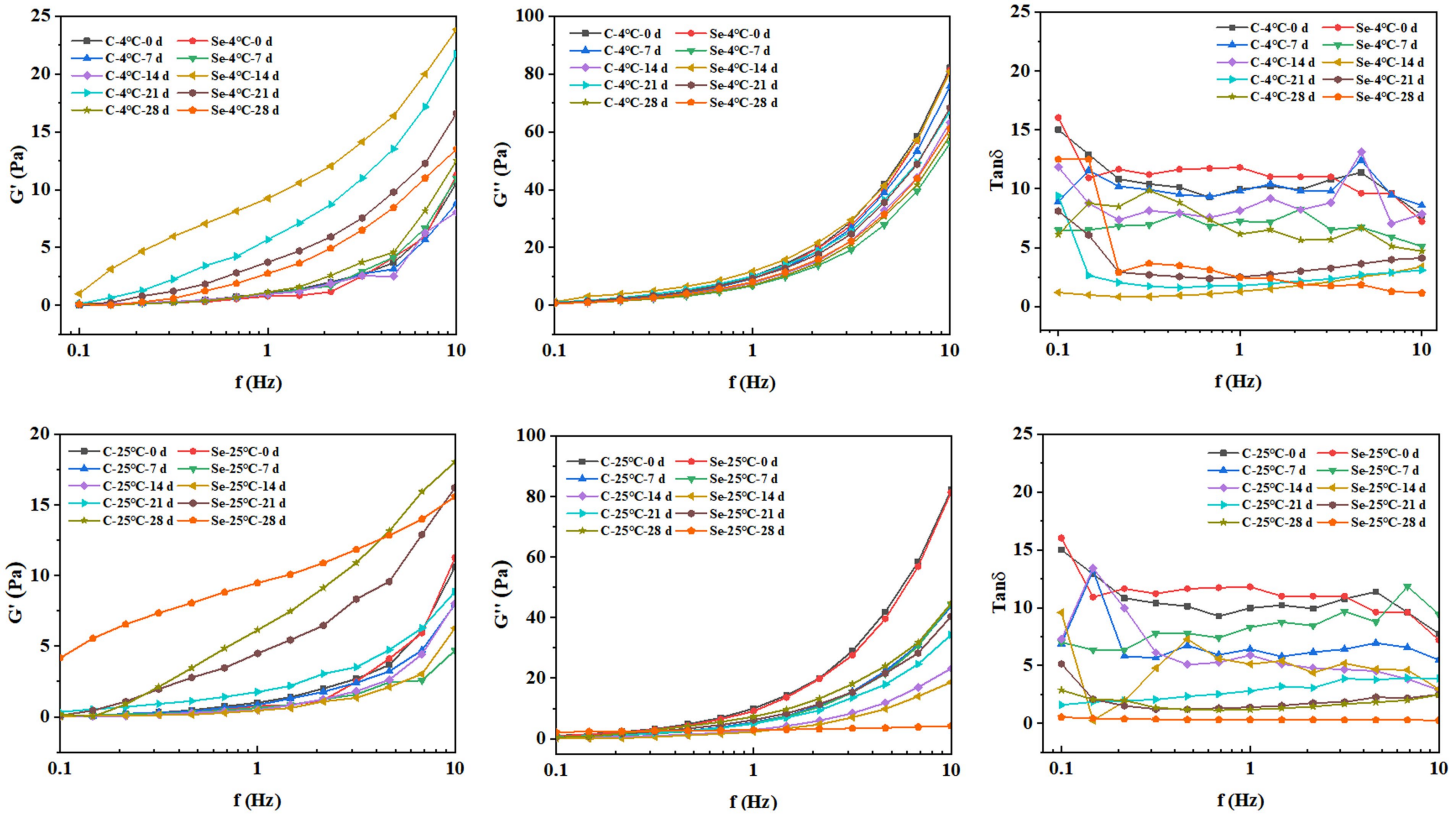
Changes in *G*′, *G*″, and tan*δ* of egg yolk during storage at 4°C and 25°C.

The viscoelasticity of the sample plays an important role in the processing characteristics of egg yolk. By measuring the changes in *G*′ and *G*″ of egg yolk samples at different frequencies, a linear relationship between oscillation frequency and modulus was constructed to study the effect of selenium-rich treatment on the internal structure and stability of egg yolk samples during storage. *G*′ reflects the solid part of the system’s attributes. *G*″ and reflect the liquid’s characteristics in the system (34). As shown in Figure 6, with increasing storage time, the overall *G*′ of the Se-group and C-group shows an increasing trend, while *G*″ shows a decreasing trend. The results indicate that the storage process leads to the conversion of viscous components into elastic components in egg yolk, which is consistent with the results of apparent viscosity. Under the same storage temperature and time, the overall *G*′ of the Se-group was greater than that of the C-group, indicating that SeMet has a significant frequency dependence and can cause changes in the structural characteristics of egg yolks, increase the repulsive force between egg yolk components, and improve the stability of egg yolks. In addition, the tanδ values of all egg yolk samples were greater than 1, which is consistent with the results reported by Primacella et al. (35), indicating that changes in selenium-rich treatment and storage conditions do not alter the fluid state of the egg yolk sample.

### 3.7. Change in gel strength

As one of the important functional properties of egg yolk, the formation mechanism of gels is closely related to the various and complex proteins in egg yolk. At the same time, the gel properties of egg yolk also determine the texture, sensory and flavor of egg yolk products (36). Table 1 shows the effects of different storage days at 4°C and 25°C on the hardness, springiness, and chewiness of the C-group and Se-group. The gel hardness of the C group and Se group decreased significantly (*p* < 0.05) with storage time, and the degree of decrease in the Se group was lower than that in the C group. The gel springiness and chewiness of egg yolks in the C-group and Se-group showed an increasing trend with increasing storage time, which may be due to the high lipid content in egg yolks. With the extension of storage time, the lipid content may slightly decrease due to various factors, resulting in the reduction of oil in the whole gel structure, making the structure relatively hard. However, the overall chewiness of the Se group is smaller than that of the C-group, indicating that the lipid structure of the Se-group is softer. Generally, selenium-rich eggs can to some extent inhibit the deterioration of egg yolks during storage.

**Table 1:** Changes in the physical properties of egg yolk stored at 4°C and 25°C.

Time (d)	Group	4°C			25°C		
		Hardness (g)	Springiness	Chewiness	Hardness (g)	Springiness	Chewiness
0	C	2159.05 ± 12.59^a^	0.29 ± 0.03^b^	461.92 ± 9.45^d^	2159.05 ± 12.59^a^	0.29 ± 0.03	461.92 ± 9.45^c,d^
	Se	1929.70 ± 7.19^b,c,*^	0.28 ± 0.01^b^	465.78 ± 2.53^c^	1929.70 ± 7.19^a,*^	0.28 ± 0.01^b^	465.78 ± 2.53^b^
7	C	2058.92 ± 31.12^b^	0.35 ± 0.02^a,b^	568.39 ± 7.75^c^	1920.50 ± 12.12^b^	0.34 ± 0.07	480.35 ± 5.97^c^
	Se	2015.41 ± 19.57^a^	0.34 ± 0.04^a,b^	525.45 ± 15.94^b,*^	1849.33 ± 37.70^b^	0.34 ± 0.05^a,b^	458.49 ± 22.02^b^
14	C	1962.83 ± 10.64^c^	0.41 ± 0.04^a^	577.09 ± 1.57^c^	1829.04 ± 6.19^c^	0.37 ± 0.02	519.40 ± 1.92^b^
	Se	1950.75 ± 5.14^b^	0.41 ± 0.06^a^	528.86 ± 2.31^a,b,*^	1703.96 ± 18.93^c,*^	0.39 ± 0.07^a^	425.33 ± 9.56^c,*^
21	C	1917.53 ± 77.75^c^	0.41 ± 0.05^a^	599.34 ± 2.66^b^	1687.04 ± 30.55^d^	0.35 ± 0.02	446.48 ± 37.26^d^
	Se	1892.84 ± 45.31^c^	0.42 ± 0.06^a^	531.49 ± 11.93^a,b,*^	1462.71 ± 81.46^d,*^	0.36 ± 0.01^a,b^	490.36 ± 28.64^b^
28	C	1492.55 ± 3.26^d^	0.41 ± 0.08^a^	660.14 ± 1.52^a^	1516.21 ± 13.55^e^	0.36 ± 0.07	814.20 ± 1.12^a^
	Se	1446.58 ± 22.88^d^	0.43 ± 0.08^a^	546.68 ± 10.72^a,*^	1304.32 ± 6.12^e,*^	0.34 ± 0.07^a,b^	722.31 ± 15.25^a,*^

The values in the table represent the average of three measurements, and ± represents the standard deviation. a-e indicate the significance of C-group egg yolks stored at 4°C and 25°C for different storage times and capital letters indicate the significance of Se-group egg yolks stored at 4°C and 25°C for different storage times (*p* < 0.05). * represents the significance between the C-group and Se-group under the same storage time (*p* < 0.05).

### 3.8. Changes in gel water distribution

**Figure 7:**
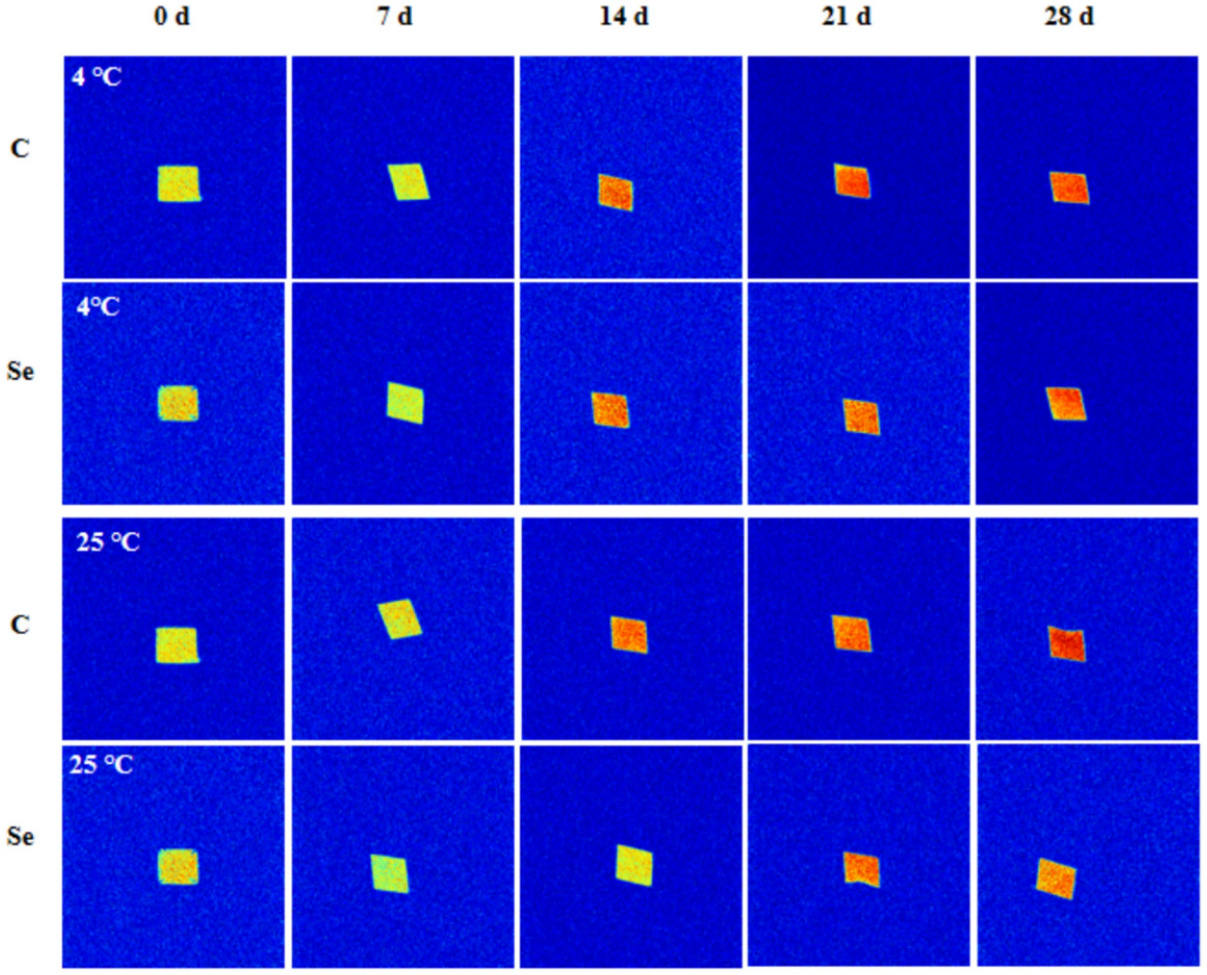
Imaging changes of egg yolk gel moisture at during storage at 4°C and 25°C.

MRI can intuitively show the water distribution and internal structure changes of egg yolk gel samples. The brightness of the hydrogen proton image is positively correlated with the water content in the gel (37). The hydrogen proton density image of egg yolk gel is shown in Figure 7. With increasing storage time, the MRI image of egg yolk gel gradually approaches red from light yellow, indicating that the water content in the gel network structure increases, which is related to the enhanced mobility of free water. Under the same conditions, compared with the C-group, the color of the MRI image of Se-group egg yolk gel samples after 14 days of storage was weaker than that of C-group samples, which confirmed that SeMet improved the water retention of egg yolk gel.

### 3.9. Changes in surface tension

**Figure 8:**
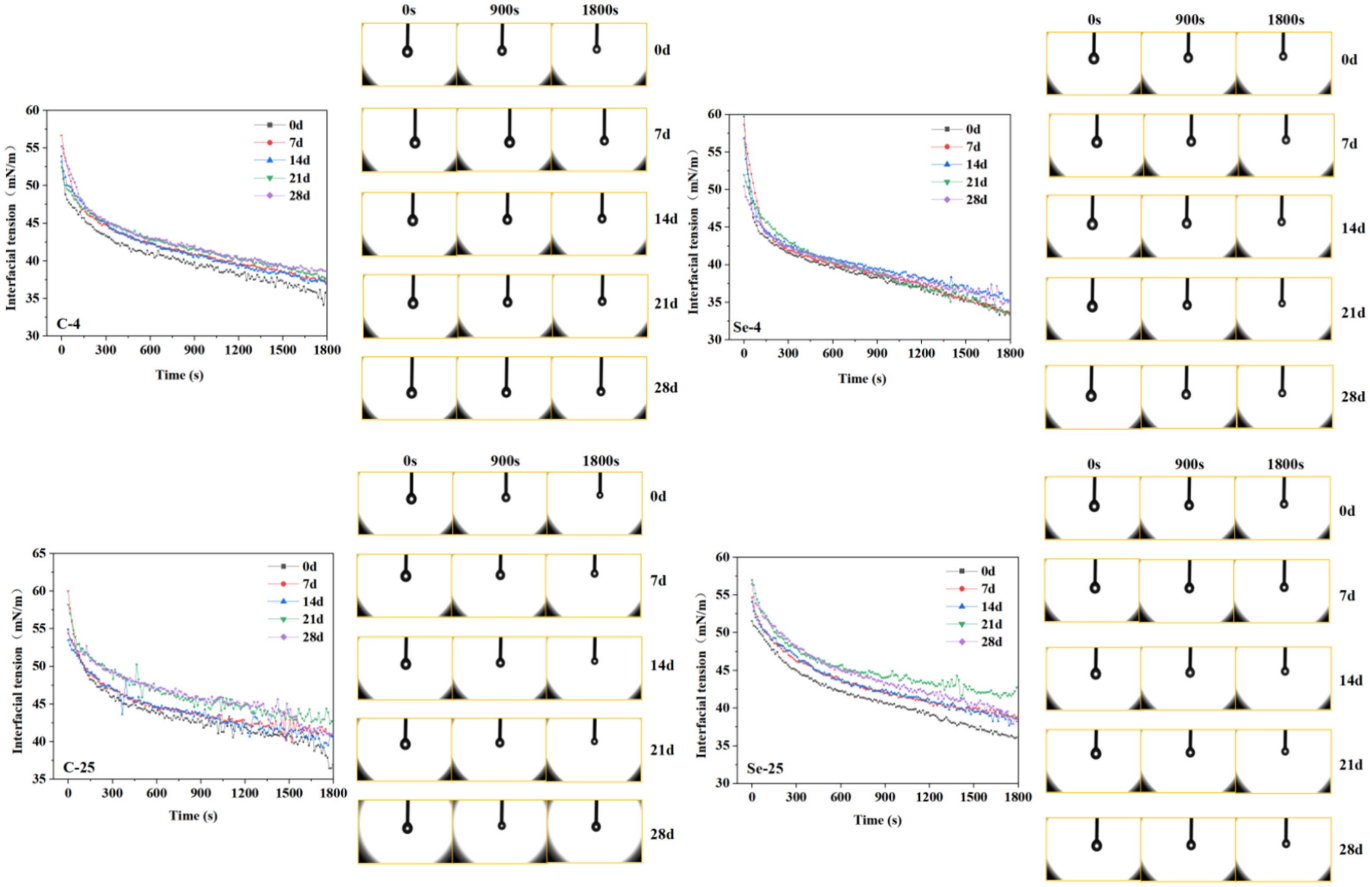
Interface tension and adsorption diagram of egg yolk during storage at 4°C and 25°C.

Egg yolk is widely used as a natural food emulsifier in food processing due to its excellent emulsifying properties (38). Therefore, evaluating the changes in egg yolk emulsifying properties can help improve its application range in the food industry. The adsorption and kinetic properties of proteins at the oil–water interface are usually reflected through interfacial tension, which generally decreases with increasing surface interaction. Figure 8 shows that with the extension of adsorption time, the interfacial tension shows a gradually decreasing trend, and during storage, the interfacial tension shows a gradually upward trend. This explains why storage can cause a decrease in emulsifying properties. The water in the egg yolk gradually increases during storage, and the phenomenon of egg yolk thinning is more obvious. The dry matter content is relatively low, resulting in a decrease in emulsifying ability. In contrast, the emulsifying properties of the egg yolk in the Se-group showed relatively small changes during storage, which is closely related to the protective effect of the eggshell. The better protective effect inhibits the thinning progress of the egg yolk, making its dry matter content relatively stable, which is conducive to maintaining emulsifying properties. The interface tension of the 4°C-storage group showed a slower upward trend, while 25°C had a relatively faster upward trend. Among them, the C-group at 25°C showed the most obvious upward trend with the extension of storage time. This phenomenon further indicates that the Se-group eggshells have a certain protective effect on egg yolk liquid, and this result also proves that low-temperature storage is beneficial for reducing the functional loss of egg yolk liquid.

### 3.10. Changes in antioxidant capacity

Egg yolk has a high lipid content, and lipid oxidation occurs during storage. The study found that during the storage of eggs, the content of polyunsaturated fatty acids (PUFAs) decreased, and the content of monounsaturated fatty acids (MUFAs) and saturated fatty acids (SFAs) increased, which significantly reduced the nutritional and edible value of eggs. The structure and function of other components, particularly proteins in eggs, can be affected by lipid oxidation products, affecting the quality and functional characteristics of stored eggs (14, 39). As a result, the lipid composition of egg yolks undergoes changes as a result of oxidation and hydrolysis, which significantly affects the nutritional value and quality of eggs.

Selenium plays a crucial role in maintaining redox balance and can resist oxidative stress. Adding selenium to the diet can significantly increase GPX levels and T-AOC, and reduce MDA content in the serum of laying hens (40). To investigate the antioxidant capacity of eggs, we measured MDA, SOD, GPX, T-AOC, and CAT to evaluate antioxidant performance. Table 2 shows that as the storage time increased, the MDA content in the Se-group and C-group increased (*p* < 0.05). Meanwhile, the rate of increase in the Se-group was lower than that in the C-group, and the rate of increase under 4°C storage conditions was lower than that at 25°C. SOD, GPX, T-AOC, and CAT in the two groups decreased significantly (*p* < 0.05), while the rate of decline in the Se-group was slow. GPx is one of the important enzymes that resist peroxides. During the production and metabolism of livestock and poultry, peroxides are produced, causing cell degeneration or necrosis. GPx can also catalyze the reduction of glutathione, reducing harmful peroxides in livestock and poultry to harmless hydroxyl compounds. By decomposing 02, reducing reactive oxygen species and inhibiting the formation of free radicals can protect the integrity of cell membrane structure and normal body function from oxidative damage and interference (41). SOD is crucial for maintaining the body’s balance between oxidation and antioxidant activity, which is able to eliminate superoxide anion radicals (14). Both CAT and GPX can promote the decomposition of hydrogen peroxide, preventing it from further producing highly toxic hydroxyl radicals (42). Zhao et al. found that adding selenide glucose to the basic diet can improve the activity of glutathione peroxidase and total antioxidant capacity in eggs of Hailan brown laying hens (43). Yeast selenium can increase T-AOC activity in green shell egg yolk, reduce MDA content, and significantly increase glutathione peroxidase activity in egg yolk (44). The results indicate that SeMet can enhance the antioxidant capacity of egg yolk and weaken its lipid peroxidation. This may be due to the increased activity of enzymes such as selenium and GPX in eggs, which inhibit lipid peroxidation to enhance the antioxidant capacity of eggs, indicating that SeMet intervention can extend the shelf life of eggs.

**Table 2:** Changes in antioxidant capacity of egg yolk stored at 4°C and 25°C.

Time (d)	Group	4°C					25°C				
		MDA (nmol/mgprot)	SOD (U/mgprot)	GPX (U/mgprot)	T-AOC (unit/gprot)	CAT (U/mgprot)	MDA (nmol/mgprot)	SOD (U/mgprot)	GPX (U/mgprot)	T-AOC (unit/gprot)	CAT (U/mgprot)
0	C	0.37 ± 0.08^b^	518.31 ± 51.62^a^	1217.03 ± 99.01^a^	1.98 ± 0.31^a^	790.29 ± 29.60^a^	0.37 ± 0.08^d^	518.31 ± 51.62^a^	1217.03 ± 99.01^a^	1.98 ± 0.31^a^	790.29 ± 29.60^a^
	Se	0.21 ± 0.04^c^	911.53 ± 28.87^a,*^	1312.87 ± 85.81^a^	2.64 ± 0.12^a^	1463.61 ± 136.13^a,*^	0.21 ± 0.04^c^	911.53 ± 28.87^a,*^	1312.87 ± 85.81^a^	2.64 ± 0.12^a^	1463.61 ± 136.13^a,*^
7	C	0.38 ± 0.03^b^	472.44 ± 73.70^a^	1064.53 ± 90.92^b^	1.41 ± 0.19^b^	451.82 ± 22.98^b^	0.63 ± 0.06^d^	473.73 ± 60.27^a^	886.18 ± 82.52^b^	1.21 ± 0.45^b^	209.72 ± 18.86^b^
	Se	0.34 ± 0.04^c^	830.60 ± 82.81^a,b,*^	890.21 ± 70.64^b^	1.93 ± 0.03^b,*^	1049.74 ± 197.82^b,*^	0.24 ± 0.04^c,*^	928.25 ± 35.56^a,*^	847.59 ± 30.81^b^	1.66 ± 0.04^b^	543.57 ± 51.12^b,*^
14	C	0.68 ± 0.09^b^	347.19 ± 21.70^b^	667.73 ± 60.12^c^	1.04 ± 0.18^c^	210.66 ± 20.24^c^	1.80 ± 0.18^c^	228.38 ± 11.77^b^	424.67 ± 3.25^c^	0.51 ± 0.14^c^	108.88 ± 8.07^c^
	Se	0.48 ± 0.07^c,*^	629.38 ± 36.06^b,c^	872.50 ± 12.67^b,*^	1.31 ± 0.03^c^	939.64 ± 61.68^b,*^	0.34 ± 0.11^c,*^	447.75 ± 35.97^b,*^	670.48 ± 43.52^c,*^	0.97 ± 0.07^c,*^	224.38 ± 31.85^c,*^
21	C	3.90 ± 0.31^a^	239.42 ± 18.32^c^	346.04 ± 6.67^d^	0.68 ± 0.11^d^	96.21 ± 2.60^d^	9.39 ± 0.29^b^	112.88 ± 13.81^c^	139.06 ± 7.83^d^	0.27 ± 0.09^c^	64.68 ± 3.70^d^
	Se	1.23 ± 0.04^b,*^	480.42 ± 12.07^c,d,*^	729.00 ± 56.57^c,*^	0.96 ± 0.20^d^	412.54 ± 13.54^c,*^	3.07 ± 0.57^b,*^	349.24 ± 5.89^c,*^	633.91 ± 27.50^c,*^	0.79 ± 0.16^c,*^	59.14 ± 3.84^d^
28	C	12.80 ± 0.64^a^	101.72 ± 12.37^d^	77.44 ± 3.42^e^	0.55 ± 0.11^d^	42.80 ± 2.68^e^	17.82 ± 0.99^a^	46.53 ± 10.12^c^	114.52 ± 3.32^d^	0.12 ± 0.04^c^	22.48 ± 2.68^e^
	Se	6.56 ± 0.37^a,*^	447.45 ± 0.68^d,*^	633.91 ± 27.50^c,*^	0.71 ± 0.10^e^	276.94 ± 7.41^c,*^	9.12 ± 0.88^a,*^	290.47 ± 10.40^d,*^	585.27 ± 58.43^c,*^	0.51 ± 0.14^d,*^	56.42 ± 2.57^d,*^

The values in the table represent the average of three measurements, and ± represents the standard deviation. a-e indicate the significance of C-group egg yolks stored at 4°C and 25°C for different storage times and capital letters indicate the significance of Se-group egg yolks stored at 4°C and 25°C for different storage times (*p* < 0.05). * represents the significance between the C-group and Se-group under the same storage time (*p* < 0.05).

### 3.11. Correlation analysis

**Figure 9:**
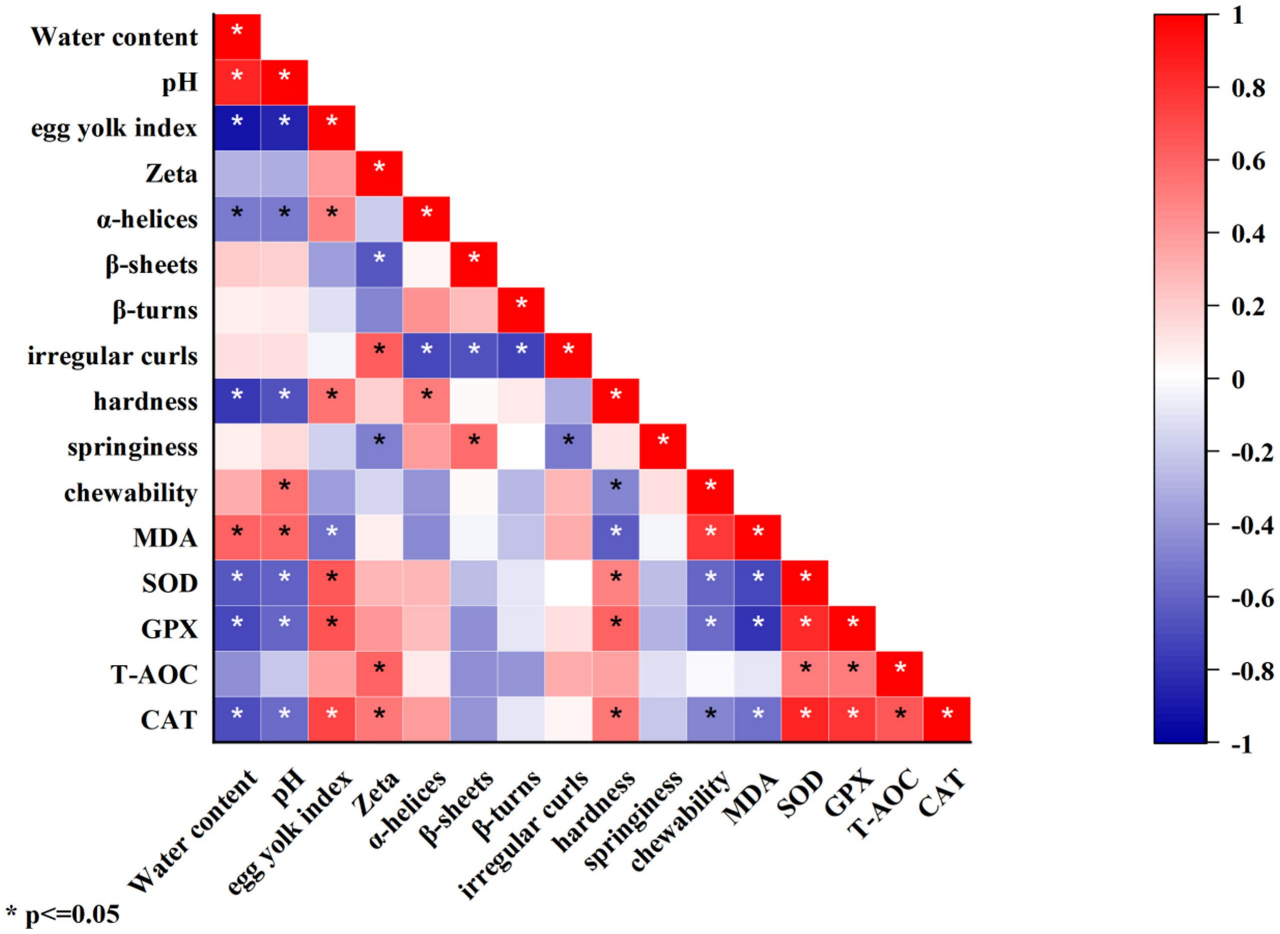
Correlation analysis between different indicators during storage.

The correlation analysis between physical–chemical indicators, structural characteristics, and functional characteristics during storage is shown in Figure 9. Using Pearson for correlation analysis, the correlation coefficient (*ρ*) range is −1 to 1, and ρ approaching 1 indicates a higher degree of correlation between the two variables. 0 < *ρ* < 0.2 represents extremely low correlation, 0.2 < *ρ* < 0.4 represents low correlation, 0.4 < *ρ* < 0.6 represents moderate correlation, 0.6 < *ρ* < 0.8 represents high correlation, and 0.8 < *ρ* < 1.0 represents extremely high correlation. The results showed that water content was significantly (*p* < 0.05) extremely highly positively correlated with pH, highly positively correlated with MDA, extremely highly negatively correlated with egg yolk index, and highly negatively correlated with CAT, GPX, SOD, and gel hardness. The pH value was significantly (*p* < 0.05) highly positively correlated with MDA, extremely highly negatively correlated with the egg yolk index, and highly negatively correlated with SOD and gel hardness. The egg yolk index was significantly (*p* < 0.05) highly positively correlated with CAT, GPX, and SOD. Zeta was significantly (*p* < 0.05) highly positively correlated with T-AOC and irregular curls and highly negatively correlated with β-sheets. α-helices, β-sheets, and β-turns were significantly (*p* < 0.05) highly negatively correlated with irregular curls. Gel hardness was significantly (*p* < 0.05) highly positively correlated with GPS and highly negatively correlated with MDA. Gel chewiness was significantly (*p* < 0.05) highly positively correlated with MDA. MDA was significantly (*p* < 0.05) highly negatively correlated with GPX and SOD. SOD and CAT were significantly (*p* < 0.05) extremely highly positively correlated with GPX. GPX, and T-AOC were significantly (*p* < 0.05) highly positively correlated with CAT.

## 4. Conclusion

This study confirms that the addition of methionine selenium can slow down the degree of egg yolk deterioration during storage. Selenium rich methods can reduce the migration of water in egg yolks during storage. Selenium-rich methods will not affect the fluid state of egg yolks during storage but can slow down the increase in pH value of egg yolks and the decrease in absolute values of apparent viscosity and Zeta potential, maintaining the original quality of egg yolks. In addition, selenium enrichment can also improve the functional properties of egg yolk during storage, improve the water retention of egg yolk gel, reduce the hardness and chewiness of egg yolk gel, and improve the antioxidant capacity of egg yolk, which is manifested in reducing the increase rate of MDA content, the decline rate of SOD, GPX, T-AOC, CAT, and slowing down the reduction of egg yolk emulsification. Besides, selenium-rich treatment can prevent the transition of egg yolk proteins to disorder and reduce the maximum fluorescence intensity of endogenous fluorescence spectra. In summary, the addition of methionine selenium can improve the strength of egg yolk film and enhance the storage stability of egg yolk.

## Data availability statement

The raw data supporting the conclusions of this article will be made available by the authors, without undue reservation.

## Author contributions

DC: investigation and writing. YL: software and writing. SX: conceptualization, funding acquisition, validation, and review. All authors contributed to the article and approved the submittedversion.

## Funding

The study was supported by the China Agriculture Research System and the National Key Research and Development Program of China (No. CARS-35-05B).

## Conflict of interest

The authors declare that the research was conducted in the absence of any commercial or financial relationships that could be construed as a potential conflict of interest.

## Publisher’s note

All claims expressed in this article are solely those of the authors and do not necessarily represent those of their affiliated organizations, or those of the publisher, the editors and the reviewers. Any product that may be evaluated in this article, or claim that may be made by its manufacturer, is not guaranteed or endorsed by the publisher.
